# Microbial acid fermentation products: An annotated selection of World Wide Web sites relevant to the topics in *microbial biotechnology*

**DOI:** 10.1111/1751-7915.13044

**Published:** 2017-12-26

**Authors:** Lawrence P. Wackett

**Affiliations:** ^1^ Department of Biochemistry, Molecular Biology & Biophysics BioTechnology Institute University of Minnesota St. Paul MN 55108 USA

## Microbial production of organic acids

http://www.sciencedirect.com/science/article/pii/S0167779907003228

This review article provides a good overview of the numerous organic acid products that can be made by microbial fermentations and the expanding markets for those products.

## Lactic acid fermentation

https://en.wikipedia.org/wiki/Lactic_acid_fermentation

The Wikipedia page describes the lactic acid fermentation, important for foods and for the production of lactic acid for making polymers.

## Polylactic acid review

http://www.sciencedirect.com/science/article/pii/S1687850714000314

This review covers the production of lactic acid by fermentation and its recovery from fermentation broths.

## Polylactic acid: Hitachi

http://www.hitachi.com/businesses/infrastructure/product_site/ip/process/pla.html

This page highlights the polymerization process for lactic acid polymerization. Lactic acid is shown to derive from sugar derived from corn or sugar beets.

## NatureWorks

https://www.natureworksllc.com

NatureWorks is a company making polylactic acid from lactic acid that has been produced by a microbial fermentation process.

## Microbial succinic acid production

https://link.springer.com/chapter/10.1007/978-3-662-45209-7_7

Succinic acid is emerging as an important bio‐product, due to its value in the food industry and also for the chemical industry as a potential replacement for the current maleic anhydride C_4_ platform chemistry.

## Succinic acid biorefinery

https://www1.eere.energy.gov/bioenergy/pdfs/ibr_arra_myriant.pdf

This page describes a biorefinery for producing bio‐succinic acid, located in the Port of Lake Providence, Louisiana, USA. The production capacity is 30 million pounds annually.

## Biochemistry of itaconic acid production

https://www.ncbi.nlm.nih.gov/pmc/articles/PMC3572532/

This paper deals with metabolic reactions producing itaconic acid. Major microbial produces are *Aspergillus* species.

## Fermentative itaconic acid production

https://www.omicsonline.org/open-access/fermentative-itaconic-acid-production-2376-0214.1000119.php?aid=28089

This review covers itaconic acid, a platform chemical that is important in textile and pharmaceutical industries.

## Malolactic fermentation

https://en.wikipedia.org/wiki/Malolactic_fermentation

The malolactic acid fermentation is most important in the wine industry. During the process, the tart‐flavoured malic acid is transformed into the softer‐tasting product, lactic acid.

## Microbial production of citric acid

http://www.scielo.br/scielo.php?script=sci_arttext&pid=S1516-89131999000300001

This review article is on citric acid production. Citric acid is the largest organic acid fermentation product, and it is produced mainly by *Aspergillus niger* and *Candida* sp.

## Citric acid in biotechnology

https://ccj.springeropen.com/articles/10.1186/s13065-017-0251-y

This review article brings out the important point that citric acid is produced in 4 billion ton quantities by biomass fermentation processes.

## Citric acid producer *Aspergillus* genome

https://genome.jgi.doe.gov/Aspni5/Aspni5.home.html

This is the genome portal page for a major citric acid producing microorganism, *Aspergillus niger*.

## Acetic acid fermentation

https://www.omicsonline.org/scholarly/acetic-acid-fermentation-journals-articles-ppts-list.php

This page on fermentation technology is focused on acetic acid fermentation. Some relevant links are provided to articles and conference proceedings.

## Microbial propionic acid production

http://www.mdpi.com/2311-5637/3/2/21

Propionic acid is produced through microbial fermentation. It has applications in the manufacture of cosmetics and pharmaceuticals.

## Alpha‐linolenic acid production

https://www.sciencedirect.com/topics/agricultural-and-biological-sciences/alpha-linolenic-acid

Alpha‐linoleic acid is considered an essential fatty acid for humans; and so, it is sought after by the food industry.

## Gamma‐linolenic acid production

http://www.sciencedirect.com/science/article/pii/S1369703X16302789

Gamma‐linolenic acid is valued for pharmaceutical and nutraceutical applications. The present paper discusses its production using the yeast *Yarrowia lipolytica*.

